# The Earliest Lead Object in the Levant

**DOI:** 10.1371/journal.pone.0142948

**Published:** 2015-12-02

**Authors:** Naama Yahalom-Mack, Dafna Langgut, Omri Dvir, Ofir Tirosh, Adi Eliyahu-Behar, Yigal Erel, Boaz Langford, Amos Frumkin, Mika Ullman, Uri Davidovich

**Affiliations:** 1 The Fredy & Nadine Herrmann Institute of Earth Sciences, Hebrew University of Jerusalem, Edmond J. Safra Campus, Givat Ram, 91904, Jerusalem, Israel; 2 Institute of Archaeology, The Hebrew University, Mount Scopus, 91905, Jerusalem, Israel; 3 The Laboratory of Archaeobotany and Ancient Environments, The Sonia and Marco Nadler Institute of Archaeology, Tel Aviv University, 6997801, Tel Aviv, Israel; 4 The Martin (Szusz) Department of Land of Israel Studies and Archaeology, Bar-Ilan University, 5290002, Ramat-Gan, Israel; 5 Israel Cave Research Center, Department of Geography, The Hebrew University, 91905, Jerusalem, Israel; 6 McDonald Institute for Archaeological Research, University of Cambridge, Cambridge CB2 3ER, United Kingdom; New York State Museum, UNITED STATES

## Abstract

In the deepest section of a large complex cave in the northern Negev desert, Israel, a bi-conical lead object was found logged onto a wooden shaft. Associated material remains and radiocarbon dating of the shaft place the object within the Late Chalcolithic period, at the late 5^th^ millennium BCE. Based on chemical and lead isotope analysis, we show that this unique object was made of almost pure metallic lead, likely smelted from lead ores originating in the Taurus range in Anatolia. Either the finished object, or the raw material, was brought to the southern Levant, adding another major component to the already-rich Late Chalcolithic metallurgical corpus known to-date. The paper also discusses possible uses of the object, suggesting that it may have been used as a spindle whorl, at least towards its deposition.

## Introduction

Lead occurs in nature mainly as galena (lead sulphide) and cerussite (lead carbonate). Prior to the Romans, who used metallic lead extensively, objects made of lead were relatively scarce and small. Because of its high specific weight, lead was mainly used by fishermen to hold down their nets, or as filling material in bronze commercial weights. Otherwise, lead was sometimes added to copper alloys in order to improve the fluidity and lower the melting temperature, properties which facilitated casting, particularly the filling of large and complex moulds. However, first and foremost lead ores were a major source of silver, and lead was merely the by-product of silver extraction [1], [2].

Silver, which often occurs in small quantities in lead ores, was separated from the lead in the cupellation process, wherein silver-bearing lead is selectively oxidized, leaving behind silver (and gold if present), which has a lower affinity to oxygen. During the 4^th^ millennium BCE, silver first appeared, quite suddenly, over a wide area, including Mesopotamia, Iran, Anatolia and the Levant, along with evidence for cupellation in the form of litharge and other production remains (e.g., [3], [4], [5], [6], [7]). Concurrently, the number of lead objects increased, indicating that lead during the 4^th^ millennium BCE was a by-product of silver production.

**Fig 1:**
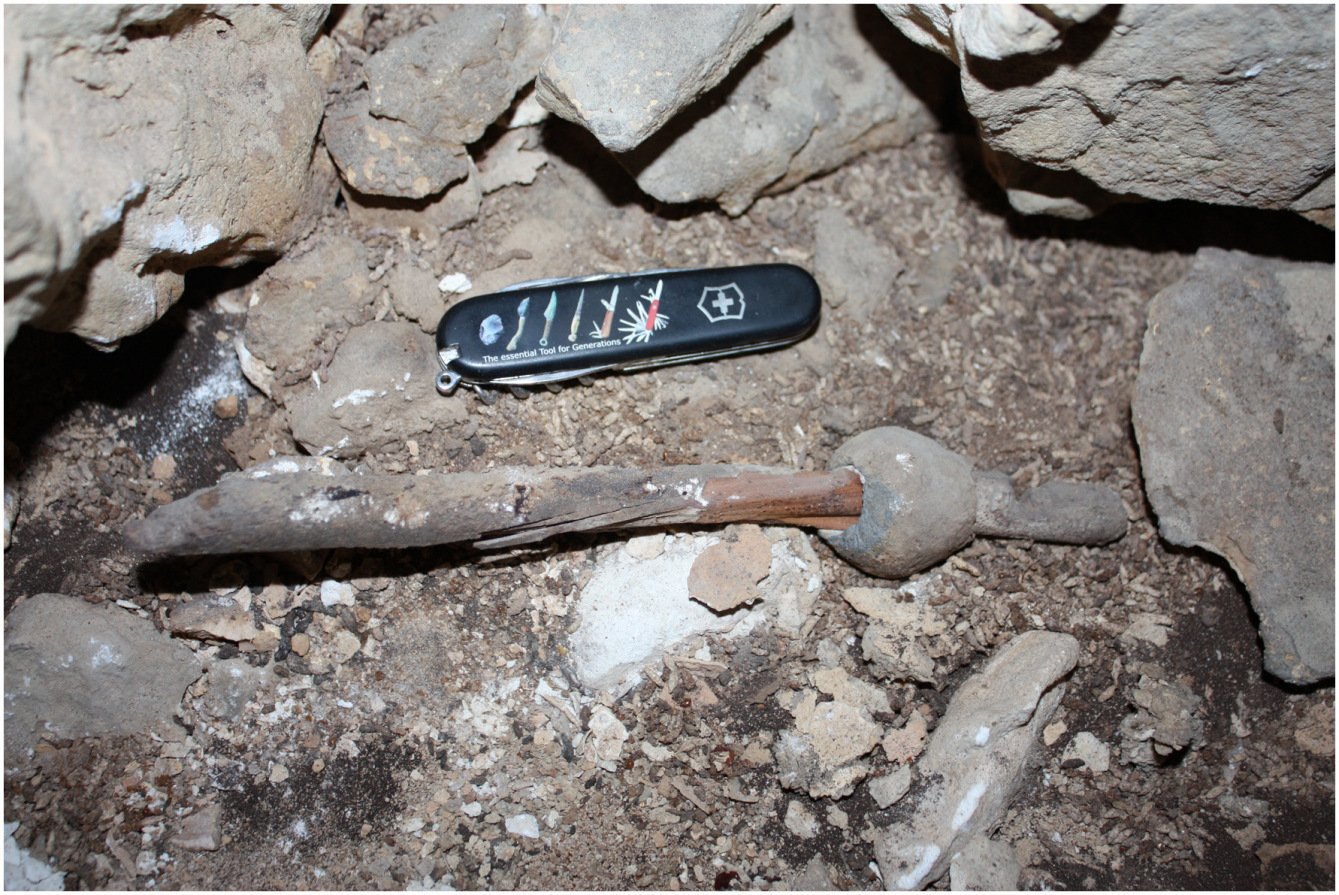
The Lead object. The lead object with shaft *insitu* at Ashalim Cave.

**Fig 2:**
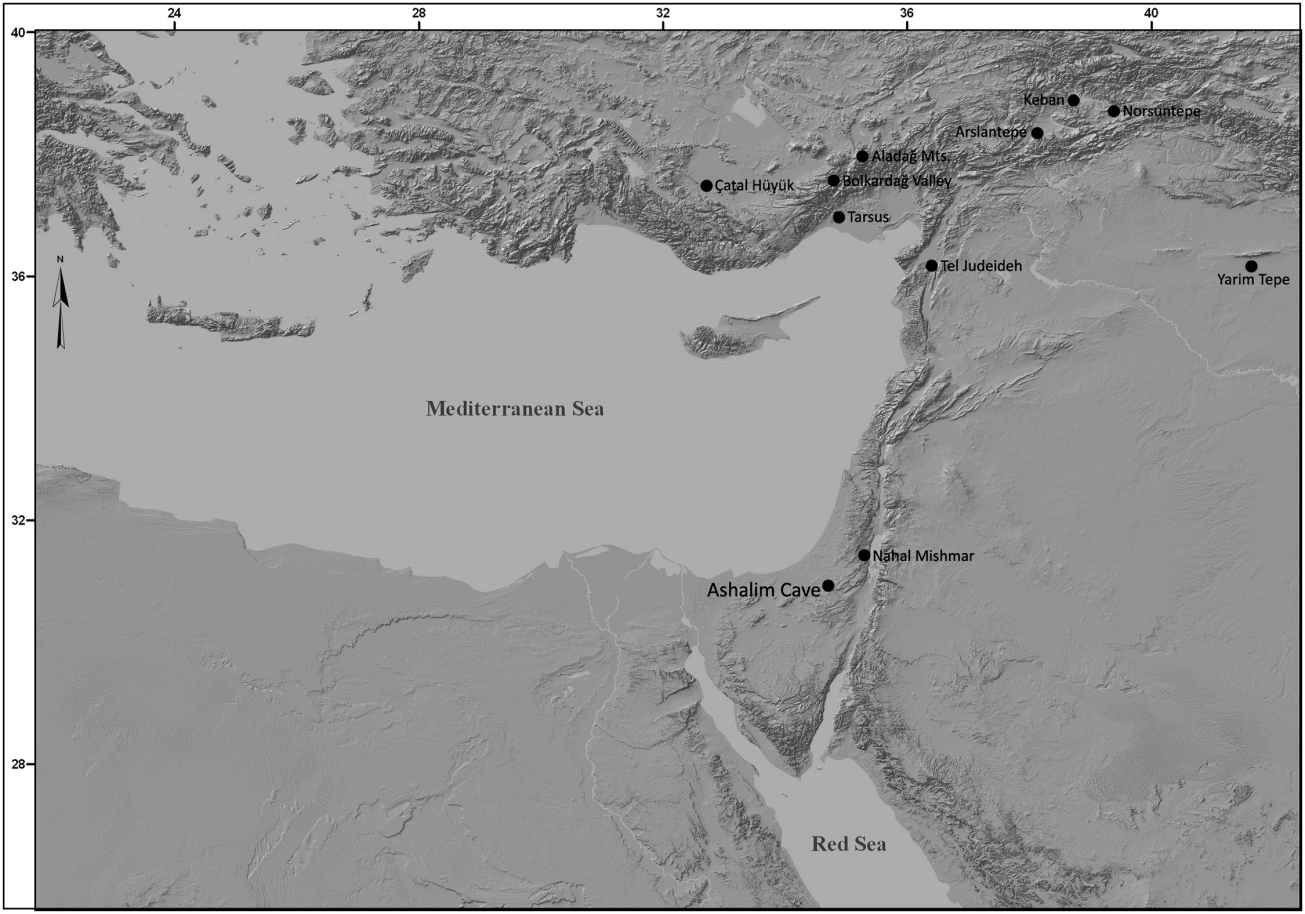
Map. The location of Ashalim Cave and additional sites that are mentioned in the text. The figure was created with ARC-GIS 10 software, based on a topographic model generated by NASA Earth Observatory (public domain).

The lead object which is the focus of this paper was found attached to an intact wooden shaft in a field survey at Ashalim Cave (Fig 1), in the northern Negev Highlands (see Fig 2). Radiocarbon dating of the shaft provided a calibrated date of ca. 4300–4000 BCE, in accordance with the ceramic and lithic typology of artefacts from the same context, which attribute the activity inside the cave to the Late Chalcolithic period [8]. The study of the Ashalim Cave object, the only pre-4^th^ millennium lead artifact ever uncovered in the Levant, sheds new light on the early metallurgy of lead, its sources, and its technological role at the formative stages of metal production in the Near East.

### Environmental and Archaeological Context

**Fig 3:**
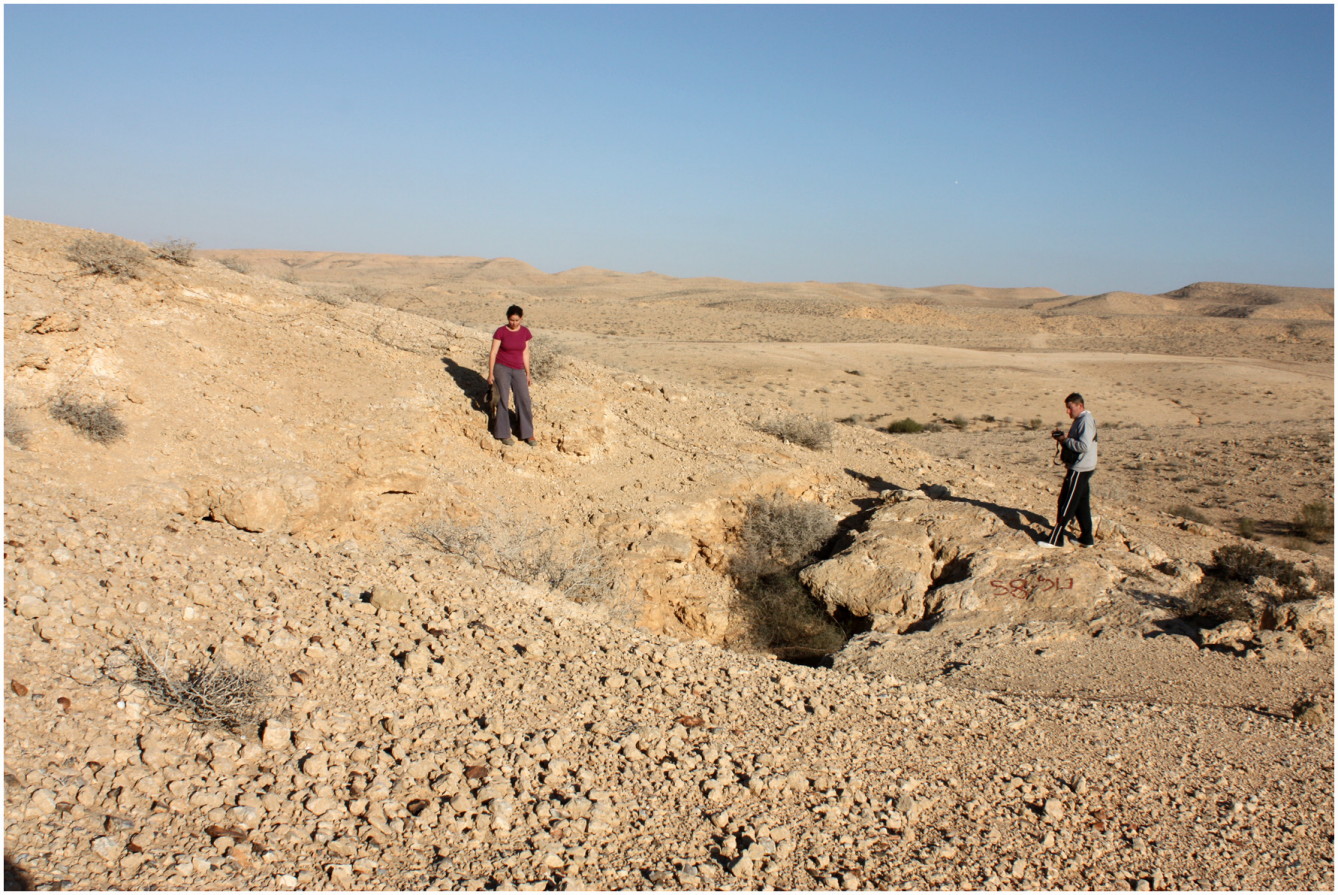
View of Ashalim Cave in its desert environment. The vertical entrance to the cave is located in front of the standing figure.

Ashalim Cave is a large complex karstic cave located on the barren slopes of Boqer Ridge, one of the northeast-trending anticlines of the northern Negev Desert (Fig 3). The cave morphology is a 3-dimensional hypogenic maze, formed >3.2 Myr ago, as indicated by the U-Pb age of its oldest speleothems [9], [10]. It is c. 30 km south of the Beer-Sheba Valley which constitutes the southernmost settled province of the southern Levant during the Late Chalcolithic. The archaeological exploration of the cave began in the early 1970s, when a short survey conducted by R. Cohen uncovered a few Late Chalcolithic vessels in an unknown location within it, as well as a few sherds from the Early and Intermediate Bronze Age [11]. During this initial survey, the inner, less accessible sections of the cave were not explored, and the cave was interpreted as a habitation site, related to the sedentary communities of the Beer-Sheba Valley.

**Fig 4:**
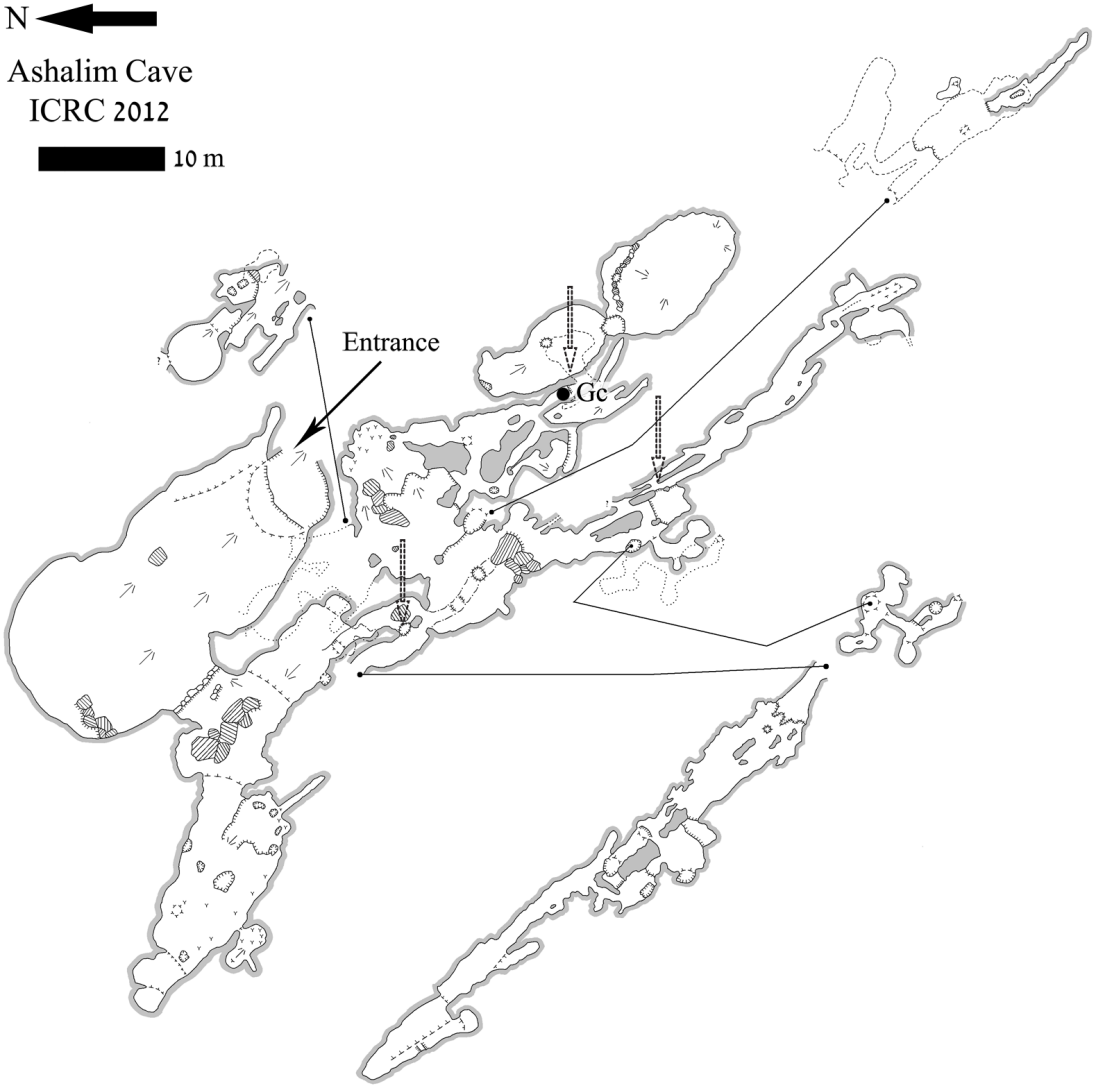
Plan of Ashalim Cave. The plan of Ashalim Cave with the location of the lead object marked by a dot.

Following a paleo-climatic study of the cave’s speleothems [9], the Israel Cave Research Center (ICRC) re-mapped the entire cave, penetrating into numerous spaces which were not previously visited (Fig 4). As archaeological remains were observed in these new areas, a detailed, systematic archaeological survey was initiated (2012). The new survey revealed that selected inner sections of the cave were reserved for mortuary activity during the Late Chalcolithic period, yielding complete and semi-complete pottery vessels, as well as a few other finds in association with human bones of two individuals [8]. It seems that the artifacts recovered by Cohen (above) are also related to the same activity, and that this was probably the sole reason for human presence in this deep, remote maze-like cave.

### Description of the Object

**Fig 5:**
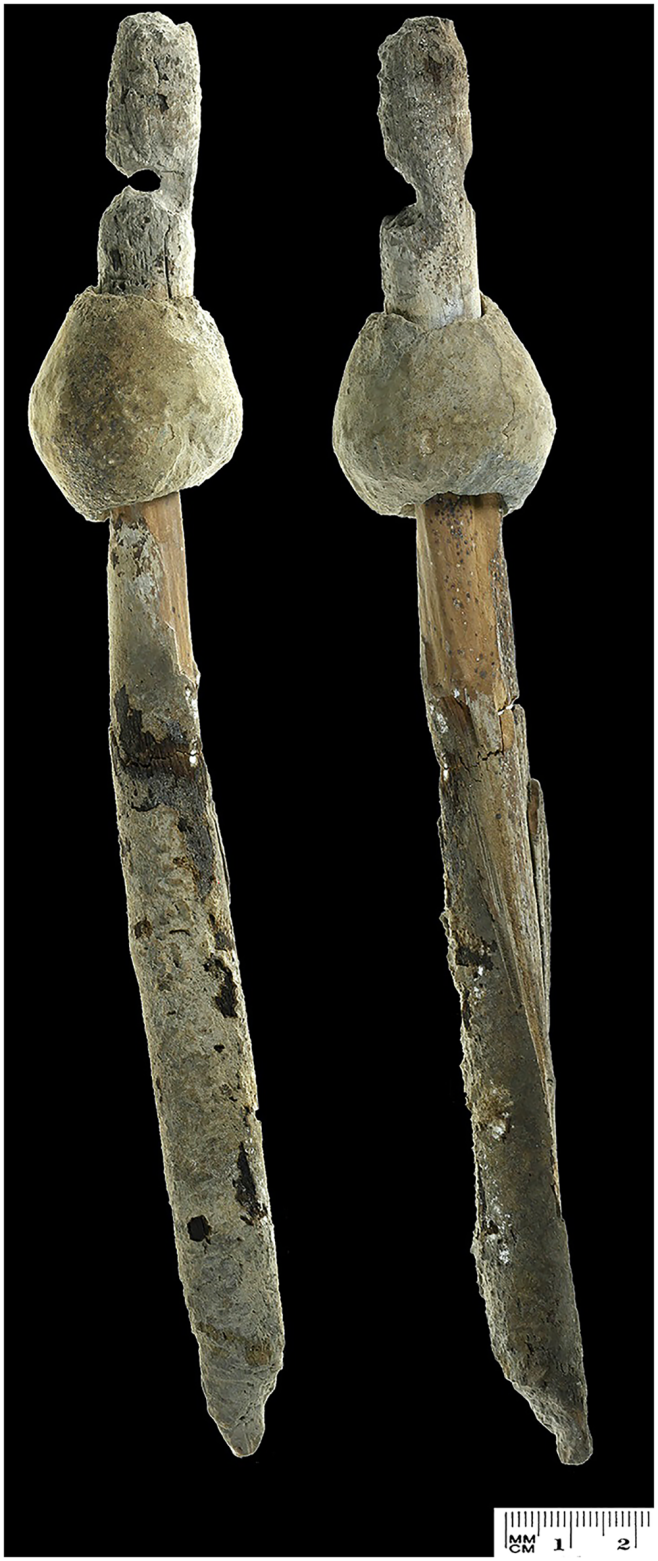
The Ashalim Cave lead object.

Further beyond the mortuary chambers, on the surface of one of the innermost narrow passages (indicated on Fig 4), a single artifact was found, composed of a 22.4 cm long wooden shaft and a perforated lead object still attached to it (Fig 5). The shaft was made of *Tamarix sp*. (Tamarisk) wood, which is a common tree genus in the Negev Desert [12]. Animal fibres (heavily degraded) extracted from the Ashalim implement may have resulted from animal activity within the cave. The lead object is bi-conical and perforated along its long axis. Its maximum height is 3.7 cm and maximum diameter is 3.6 cm, and it weighs 155.8 g. The perforation, drilled after the initial manufacture of the metal object, measures 1.6–1.8 cm in diameter; its interior is whitish in color, while the surface of the metal is grey. The metal object was positioned 4 cm from the shaft’s finished edge. Between the edge and the metal object, a 9 mm depression was cut into the shaft. Abrasion marks are visible at the bottom of the lead object, possibly as a result of use (see below).

## Methods

The Ashalim Cave object (Locus 120; Reg. No. 1068) was found during an archaeological survey conducted under the approval of the Israel Antiquities Authority, License Number S-337/2012. It is available at the conservation lab in the Institute of Archaeology, The Hebrew University of Jerusalem, Lab No. 6183. No additional permissions were required for the study of this cave, as the site is not located within the boundaries of a national park, nor within a private property. The individual in Fig 3 of this manuscript has given written informed consent (as outlined in PLOS consent form) to appear in the picture.

### Analytical Methods

**Fig 6:**
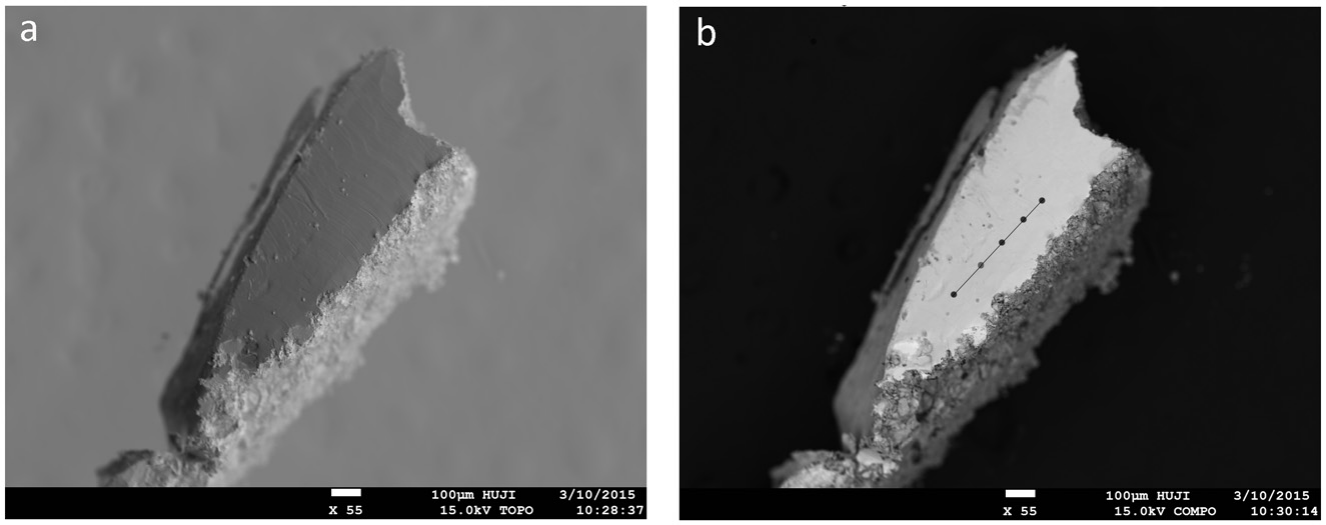
Microscopic images of the sampled area. (a) Secondary electron and (b) Back-scatter images of the freshly cut surface. The five measured points are marked on Fig 6b.

Two small samples of metal were extracted, using a scalpel, from the inner part of the perforation for chemical, isotopic and microstructure analyses. Half of one sample was mounted in epoxy; however, various polishing attempts were unsuccessful, as the soft metal absorbed crystals from the different polishing agents. The other half was analysed using a JEOL 8230 superprobe EPMA, in the Institute of Earth Sciences at the Hebrew University of Jerusalem, equipped with EDS and four wavelength-dispersive spectrometers (WDS). Beam conditions were set at 15keV and 15 nA with LaB6 Filament. All elements were analysed using silicate and oxide standards (natural minerals—spi 53) and PRZ correction procedure. The piece was cut in order to create a relatively flat fresh surface and immediately inserted into the chamber without mounting and polishing, in order to avoid oxidation and contamination (Fig 6). The freshly cut surface was measured at five different points, 1 micron in diameter each (Fig 6b). The limit of detection (LOD) was calculated for each element based on the specific conditions that prevailed during the measurement, and is presented in Table 1.

**Table 1:** Normalized trace element concentrations (ppm) measured by WDS.

	Ag	Cu	Ni	Ti	Fe	Zn	Co
Minimum	0	0	0	20	0	0	140
Maximum	760	320	150	250	180	290	540
Average ppm	290	60	30	130	50	90	390
STD	400	140	70	80	80	130	230
LOD	≤86	≤90	≤49	≤46	≤49	≤94	≤64

A small chip was removed from the object for lead isotope analysis (LIA). In this method, lead isotope abundance ratios, which do not modify during the smelting and re-melting process, are used to relate metal objects to their parent ore sources (for discussion and bibliography see, [13], [14], p. 31–38, [15], [16]).

The sample (22.7mg) was dissolved in nitric acid and diluted to a concentration of 100 ppb. The solution was analysed using a Neptune plus multi-collector ICP-MS. Thallium was used for mass-bias correction. Replicate measurements of ^208^Pb/^206^Pb, ^207^Pb/^206^Pb and ^204^Pb/^206^Pb ratios of SRM-981 standard over the course of this study yielded the following values: ^208^Pb/^206^Pb = 2.16610 ± 0.00007, ^207^Pb/^206^Pb = 0.91453±0.00003, ^204^Pb/^206^Pb = 0.059066± 0.000001, (2s), n = 3. These results are in good agreement with previously reported data (see, [17]).

## Results

### Chemical Composition

The results of the chemical analysis show that the object was made of almost pure metallic lead, comprising 99.9±0.045 wt% Pb. The five measured points contained varying concentrations (0–760 ppm) of trace elements presented in Table 1. Co exceeded the limit of detection (LOD) in all five points (140–540 ppm), indicating that the object was made of smelted lead. Other trace elements such as Ag, Cu, Ni, Fe and Zn were detected only in some of the points, but in concentrations that exceeded the LOD. Other elements, including As, Bi, S, Sb and Au were not detected at all.

### Lead Isotope Analysis

**Fig 7:**
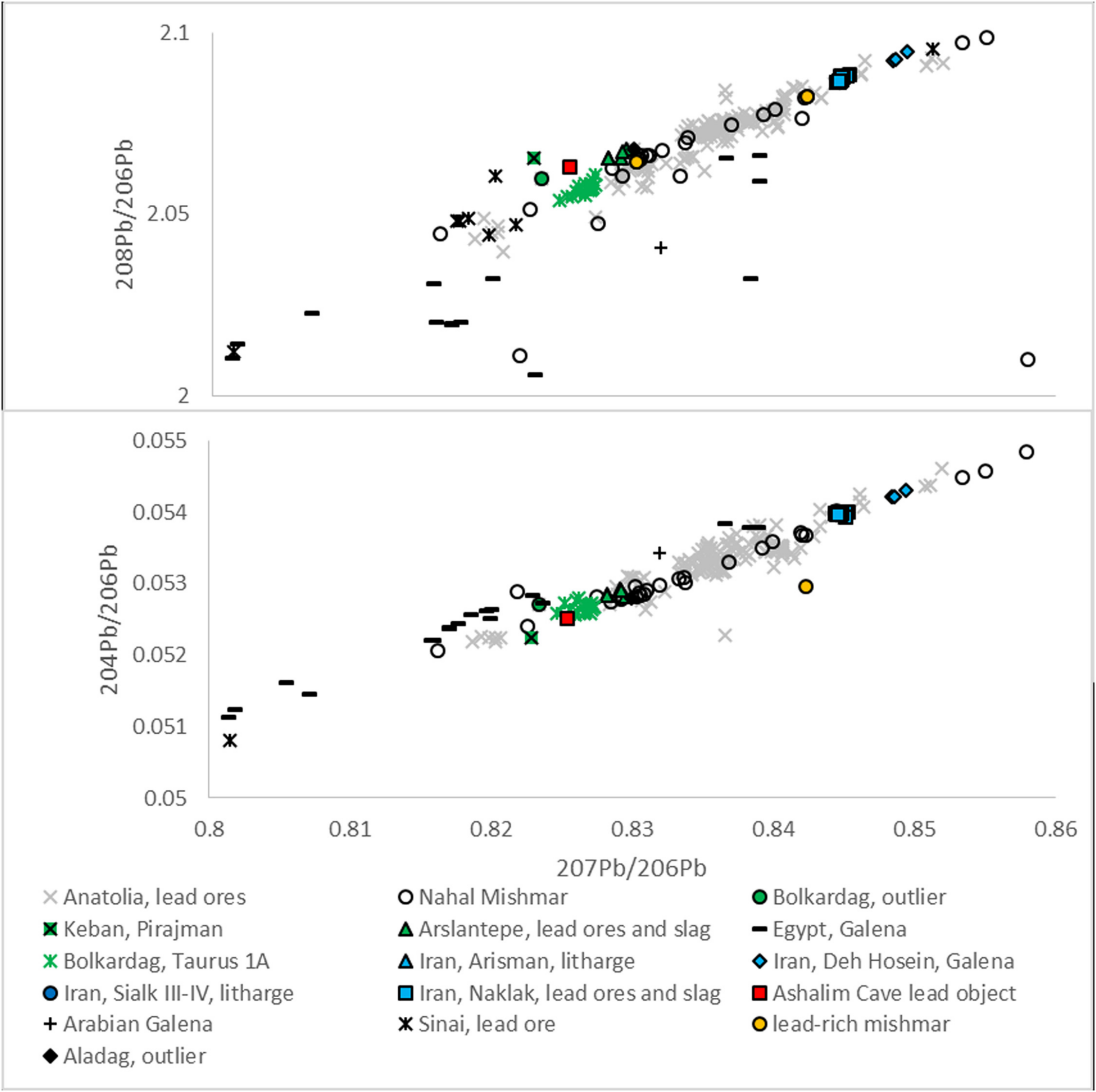
Results of lead isotope analysis. Lead isotope ratios of the Ashalim Cave lead object plotted against ores from selected regions. Details and references are provided in the text.

Lead isotope analysis of the Ashalim Cave object yielded the following values: ^208^Pb/^206^Pb = 2.0631±0.00002, ^207^Pb/^206^Pb = 0.82541±0.000009, ^204^Pb/^206^Pb = 0.052515±0.000002. These were compared with the limited lead isotopic data that is available for lead ores from neighbouring regions, such as Sinai [18], the Eastern Desert of Egypt [19], and Iran [20], [21], [22], [23], showing inconsistency with these regions. Comparison with lead ores from Anatolia [4], [24], [25], [26], [27], [28], [29], [30], [31], [32], showed that the lead isotope ratios of the Ashalim Cave object are broadly similar to lead ores from the Taurus mountain range in this region (Fig 7). These include Taurus 1A group from Bolkardağ and an outlier (AON070) from the same deposit, as well as an outlier from Aladag (AON457). One outlier from the Pirajman mine at Keban in the uppermost Euphrates Valley (TG-180B), also has similar isotopic values [26]. Several lead ore and slag fragments from Arslantepe in eastern Anatolia, Stratum VII, dated to the mid-4^th^ millennium BCE, are also broadly consistent with the ore samples listed above and with Ashalim Cave object [33].

## Discussion

### The Significance of Early Lead Smelting

The chemical analysis of the Ashalim Cave object indicates that the lead was likely to have been smelted from a relatively pure ore. Prior to the 4^th^ millennium BCE, only a handful of lead objects made of metallic lead have been reported from datable contexts, all from northern Mesopotamia and eastern Anatolia. These include a bracelet from Yarim Tepe I found in a context dated to c. 5,700 BCE [34], and a small conical object from Arpachiyeh dated to the 5^th^ millennium BCE ([35], p. 104, [36]). However, both objects were never inspected for their metallurgical properties, and their identification as being the products of lead smelting is conjectural, and is based on the rarity of native lead and the relative ease of lead smelting, which can be performed at temperatures below 800°C ([37], [38], p. 75–76). Several beads from Level IX at Çatal Höyük (7^th^ millennium BCE) were published as smelted lead ([39], p. 217–219), but are currently thought to have been shaped from galena [40]. Thus, it appears that the Ashalim Cave object, based on the concentration of trace elements such as cobalt, might be the earliest lead artifact proven so far to have been produced from smelted lead.

The Ashalim Cave object is dated to the Late 5^th^ millennium BCE. It is thus positioned between the beginning of copper smelting in the early 5^th^ millennium (e.g., [41], [42], [43], [44]), and the use of lead ores for the extraction of silver during the 4^th^ millennium BCE (see above). As such, it does not contribute to our understanding of early smelting procedures (see, [4], p. 178–179), but rather to the question of the development of silver extraction from lead ores: was regular lead smelting from lead-rich ores performed during the 5^th^ millennium BCE, a process that might have resulted in the discovery of silver few hundreds of years later? Or, alternatively, did the practice of cupellation begin earlier than previously thought? The chemical composition of the Ashalim Cave object cannot support either of the aforementioned possibilities at this stage of research, and the whole subject should be addressed when more artifacts are discovered and analysed. Meanwhile, the possibility remains, that the Ashalim Cave object being singular, represents the kind of ad hoc, ‘reinvention’, suggested by Gale and Stos-Gale ([4], p. 180), wherein lead was smelted in different unrelated circumstances.

### The Origin of The Lead

It is notable that the possible sources of the lead ore of the Ashalim Cave object all concentrate in south-central and southeastern Anatolia. The Bolkardağ valley lies 50 km north of the Mediterranean coast. It is considered the source of some of the richest argentiferous lead ore deposits in the Near East, and geologists in Turkey have reported ancient metalworking there as well as Chalcolithic settlements from as early as the 5^th^ millennium BCE [30], [45]. Since several lead objects from 4^th^ millennium BCE contexts at sites such as Tarsus and Tell Judeidah have lead isotope ratios consistent with the Taurus ores, Yener *et al*. suggested that these ore deposits were exploited as early as the 4^th^ millennium BCE [30]. The distribution of the earliest known lead objects in eastern Anatolia/northern Mesopotamia noted above, combined with the analysis of our object, point to this broad region as a source of early lead.

### The Ashalim Cave Object and the Late Chalcolithic Copper Metallurgy

The Ashalim Cave object is contemporaneous with the Late Chalcolithic extraordinary copper-based metallurgical crafts, best represented by the 400+ objects from the Cave of the Treasure in Nahal Mishmar (see Fig 2). The objects of this hoard, as well as similar objects in numerous Late Chalcolithic sites, are divided into two general groups based on typological, technological and chemical composition analyses: simple working tools (i.e., axes, adzes, awls), made of nearly-pure copper by open casting, and elaborate, “prestigious” items (maceheads, “standards”, “crowns”, etc.) made by the lost-wax casting technique of copper alloys with arsenic, antimony, and sometimes nickel and bismuth (e.g., [45], [46], [47], [48], [49], [50], [51]).

Key, who was the first to chemically analyse the copper-based items from the Nahal Mishmar hoard, reported the use of metallic lead for repairing some of the castings; he also detected lead (c. 2%) in two of the alloyed objects [46]. Tadmor *et al*., who also analysed several objects and their repairs, showed that the latter were made of the same alloy as the vessels themselves (rather than from metallic lead as suspected by Key), representing repairs carried out during the production process and not later additions [48]. Lead content between 1–3% was indeed detected in a few objects from Nahal Mishmar by Shalev and Northover and by Tadmor *et al*., corroborating Key's initial results [47], [48]. However, the low lead content alone is not necessarily an indication that lead was added to the melt as it could have originated in the parent ore. Tadmor *et al*. also showed that isotopic abundance ratios of Pb-rich items varied, suggesting that the potentially added lead could not have originated in one single source, rendering the addition of metallic lead highly unlikely ([48], p. 139–140).

Two broad origins have been suggested for the copper-based objects in the Nahal Mishmar hoard, one being the nearby Arabah Valley and the other being Anatolia and regions to its north (e.g., [48], [52]). The proximity of the latter region to the suggested origin of the lead of the Ashalim Cave object may point to common transfer routes by which raw materials were brought to the Levant, and more specifically to the south of Israel. These trade connections between the two regions were not limited to metals, as indicated for example by the import of obsidian to the Levant, which began already during the Neolithic and continued during the Late Chalcolithic (e.g., [53]). It has been shown that the copper-based items of the Late Chalcolithic southern Levant produced by the lost-wax technique were locally manufactured ([49], and see [51] with further references), pointing to the possibility that the Ashalim Cave lead object was also locally produced.

It has been noted that the Cu-As-Sb composition used for the production of the elaborate Late Chalcolithic objects in the southern Levant was also used in Arslantepe and Norsuntepe in Anatolia, albeit in a slightly later period (mid-4^th^ millennium BCE) (see, [33], [54], [55]). In fact, one of the Cu-As-Sb ore fragments (TR-8/99) that were uncovered in Arslantepe had LI abundances and chemical composition perfectly consistent with a mace (61–83) and a mace head (61–253) from the Nahal Mishmar hoard ([33], p. 53). The occurrence of lead ores and slag in Level VII at Arslantepe with lead isotope ratios broadly consistent with the lead isotope ratios of the Ashalim Cave object (see above), may be therefore significant, suggesting possibly that both sites used ores from similar sources.

### The Significance and Function of the Object

The Ashalim Cave object is unique, as no other lead objects were found in the Levant in contexts that pre-date the 4^th^ millennium BCE. Moreover, the results of the lead isotope analysis showing that it was made of lead that likely originated in Anatolia, render this object with particular significance. This may be supported by its final deposition in the deepest section of a complex maze cave, very difficult to access and used solely during the Late Chalcolithic for ritual activities related to the burial of specific individuals [8]. The connection between metals, including rare ones (e.g., [56]), and burials is well attested during the Late Chalcolithic, and is probably related to the symbolic aspects assigned to the products of these important pyro-technologies (e.g., [49], [57], [58]).

The form of the Ashalim Cave lead object recalls the typical Late Chalcolithic maceheads, which are considered to have been mostly ceremonial in function (see above). However, its weight and size places it among the smallest maceheads known from this period (compare [45], p. 130), and its composition (of lead rather than copper or stone) sets it apart from all other known maceheads. Another possible interpretation, raised by Langgut *et al*. [12], asserts that the Ashalim Cave object was a spindle, in which case the shaft would have been the spindle rod and the lead object would have been used as a whorl. This interpretation is based on the morphology of the whole artifact, including the position of the depression and the perforated metal object, which resemble high-whorl spindles, and on abrasion marks visible on the lead object, possibly the result of the spinning process. Several other wooden shafts, not logged with a whorl, were recently found in a similar context (mortuary activity in a deepest section of a maze-like cave) in Qina Cave, also located in the northern Negev and dated to the Late Chalcolithic (Davidovich *et al*. forthcoming); these were identified as spindle rods and related spinning implements based on microscopic remains of linen fibres [12]. The metal weight is, however, slightly heavier than most known whorls (usually made of stone), and could only have been used to produce rather course yarn. We may speculate that the lead object was originally used in another context, which would have made it possible to acknowledge its rarity and significance (e.g. as a macehead), before being re-used as a spindle whorl, joined to the wooden rod, at a later stage. Its eventual deposition in the deepest section of Ashalim Cave, in relation to the burial of selected individuals, serves as evidence of the symbolic significance it possessed until the final phase of its biography.
